# Prediction of hospital bed capacity during the COVID− 19 pandemic

**DOI:** 10.1186/s12913-021-06492-3

**Published:** 2021-05-18

**Authors:** Mieke Deschepper, Kristof Eeckloo, Simon Malfait, Dominique Benoit, Steven Callens, Stijn Vansteelandt

**Affiliations:** 1 Strategic Policy Cell at Ghent University Hospital, C. Heymanslaan 10, 9000 Ghent, Belgium; 2 Department of Public Health and Primary Care, Ghent University, C. Heymanslaan 10, 9000 Ghent, Belgium; 3 Department of Intensive Care Medicine, Ghent University Hospital, C. Heymanslaan 10, 9000 Ghent, Belgium; 4 Department of General Internal Medicine, Ghent University Hospital, C. Heymanslaan 10, 9000 Ghent, Belgium; 5 Department of Applied Mathematics, Computer Science and Statistics, Ghent University, Krijgslaan 281 S9, 9000 Ghent, Belgium; 6 Department of Medical Statistics, London School of Hygiene and Tropical Medicine, Keppel Street, London, WC1E 7HT UK

**Keywords:** COVID− 19, Multistate modeling, Poisson modelling, Hospital data

## Abstract

**Background:**

Prediction of the necessary capacity of beds by ward type (e.g. ICU) is essential for planning purposes during epidemics, such as the COVID− 19 pandemic. The COVID− 19 taskforce within the Ghent University hospital made use of ten-day forecasts on the required number of beds for COVID− 19 patients across different wards.

**Methods:**

The planning tool combined a Poisson model for the number of newly admitted patients on each day with a multistate model for the transitions of admitted patients to the different wards, discharge or death. These models were used to simulate the required capacity of beds by ward type over the next 10 days, along with worst-case and best-case bounds.

**Results:**

Overall, the models resulted in good predictions of the required number of beds across different hospital wards. Short-term predictions were especially accurate as these are less sensitive to sudden changes in number of beds on a given ward (e.g. due to referrals). Code snippets and details on the set-up are provided to guide the reader to apply the planning tool on one’s own hospital data.

**Conclusions:**

We were able to achieve a fast setup of a planning tool useful within the COVID− 19 pandemic, with a fair prediction on the needed capacity by ward type. This methodology can also be applied for other epidemics.

**Supplementary Information:**

The online version contains supplementary material available at 10.1186/s12913-021-06492-3.

## Background

The enormous impact of the COVID− 19 pandemic has surprised many hospitals beginning March 2020. It soon became apparent that the capacity of hospital beds was at the verge of coming under great pressure. Besides a shift of regular beds to specific COVID-19 beds with special hygiene measures, pressure on the number of beds arose primarily from the need to foresee sufficient capacity in the Intensive Care Unit (ICU). Indeed, while approximately 9 to 11% of admitted COVID-19 patients were in need of advanced life-supporting measures [1], ICU capacity was limited in terms of the number of beds, but also the number of monitoring devices, life supporting machines and specific trained personnel to provide high quality of care. In the Belgian situation, which we will consider, the number of ICU beds is on average 15.9 per 100.000 inhabitants; it is less favourable in the rest of Europe, numbering 11.5 per 100.000 inhabitants [2]. For healthcare systems, and hospitals within these systems, organizational preparedness and capacity planning was thus essential [3]. In Belgium, for instance, a Surge Capacity Plan [4] has been set up to monitor the number of occupied ICU beds and to create extra ICU beds where needed. In cases of (near) saturation, contact had to be made with the local health inspector. Moreover, so long as saturation was threatened at the national level, a certain percentage of the beds needed to be allocated and foreseen for potential COVID-19 patients (described in different phases by the government). To attain the required capacity, patients in burn units were centralized at the national level.

Hospital capacity planning is driven by complex dynamics between input, output and the number of available beds [5, 6]. In normal times, hospitals aim to achieve an optimal bed occupancy by maximizing bed occupancy while minimizing overflow, which often has a negative effect on patient outcomes [7]. However, pandemics and natural disasters typically come with a sudden influx of unforeseen patients, which almost instantly pushes the boundaries of a hospital’s capacity [8]. Frontline health care workers, directly engaged in the diagnosis, treatment, and care for patients with COVID-19, are susceptible to experience psychological burden in return, while also being at greater health risks [9]. Lack of bed capacity, scarcity in supplies and high occupancy rates further increases that burden.

In order to prevent such overflow, healthcare systems can take several measures. In China, new hospitals have been built [10], which immediately increased capacity via a larger number of available beds. However, most European countries underestimated the pandemic potential and virulence, and as such did not take such actions. In most countries, the influx in hospitals was instead reduced by means of a nationwide quarantine, measures of social distancing, hand washing, school closures, mouth mask or other activities [3]. Such measures successfully flattened the curve, decreasing the influx and therefore putting less stress on hospital capacity.

However, successfully flattening the curve means extending the duration of the pandemic, making it impossible to further postpone regular care [11]. A fragile equilibrium needs to be found between reserving a sufficient number of beds for COVID-19 cases, while also providing sufficient beds for regular, necessary care which cannot be delayed. In order to achieve such balance, predictive models can play an important role, not only to predict the number of needed beds that should be allocated to the pandemic, but also to inform the hospital on providing the right equipment and training sufficient healthcare workers for specific cases [12].

The ability to predict hospital bed capacity for different types of wards is essential for monitoring and planning purposes during epidemics, such as the ongoing COVID-19 pandemic. At the start of the pandemic, the available models were scarce. Most efforts then focused on susceptible-infected-removed (SIR) models, and variations thereof, aimed at predicting the number of positive COVID-19 cases at a national level (e.g. [13, 14]). While these provide valuable insights into the dynamics of future disease spreading in a population, we could not readily make use of those for hospital planning because they demand input on parameters, such as doubling times and social distancing measures, that were not available for our local setting at that time. Moreover, they are not designed to give detailed predictions for a specific hospital, organized by type of ward. A further limitation is that standard SIR models not accounting for cohort structure underestimate the peak of COVID-19 infectious cases and their timing [15]. General purpose simulation toolboxes, such as the (free) web application corona.simbox.ai, predict capacity building on observed trends in the number of new cases and the expected length of hospital stay observed in specific countries (data from https://www.worldometers.info/coronavirus/). While potentially more directly useful, their generic nature has the disadvantage of providing capacity predictions that are not well aligned with the regional variation in the severity of the epidemic, local treatment, triage and hospital management policies. Within the Ghent University Hospital, we have therefore set up a planning tool to predict on each day the needed capacity of different bed types over the subsequent ten-day period. Based on the tool’s predictions, the required human capacity (i.e. healthcare workers) can be assessed and the needed material can be stocked. Such capacity planning forms an essential primordial step in preparing a hospital. In particular, we will develop a data-driven prediction algorithm which makes use of daily updated hospital records to make predictions on each day, of the number of new cases that can be expected over the next 10 subsequent days, as well as how admitted cases are expected to transition during this period between different wards, as well as to discharge or death. The proposed algorithm makes use of Poisson models with smoothing splines to model the evolution in the number of new cases over time, along with multistate models [16] to describe patient transitions between multiple states (namely, wards, discharge or death). These fitted models, which are daily updated, are then used to simulate the capacity needed over the subsequent 10 days.

## Methods

### Population

The data includes all patients admitted to the University Hospital Ghent and labelled as COVID-19 patient; some of these are transferals from other hospitals. We label a patient as a COVID-19 patient, when a positive PCR test in the lab (internal or external) is present. Some patients in our dataset had already been admitted before obtaining a positive PCR test result (e.g. in the Rehabilitation department). We use data from different time points from one hospital and use as such a longitudinal study design.

### Inclusion criteria

The model is trained on all positive COVID-19 patients who were in the University Hospital Ghent before April 20, 2020. We test the model on the patients who were in the hospital between April 20, 2020 and April 27, 2020. This range of dates falls within the peak of the pandemic and refers to one of the first weeks the model was fully operational.

### Statistical modelling

The implementation of the different steps can be found in the Supplementary Appendix, along with code snippets.

### Multistate model

We use multistate modelling to model the time for current patients needed to transition to a different ward, as well as to discharge or hospital death. In particular, we model transitions between the wards Non-Covid19, Cohort, ICU Midcare, ICU Standard and ICU Ventilated. Here, Non-Covid19 includes all the wards without positive COVID-19 patients, such as the Emergency Department, but also the wards where non-COVID-19 patients stay during the pandemic. Cohort includes all wards with COVID-19 patients who need standard care. The task force decided to open a specific COVID-19 midcare unit (ICU Midcare), to better guarantee availability of Intensive Care department (ICU) beds for the most severely ill patients with a good chance of ICU survival. The ICU was further divided into unventilated critically ill patients (ICU Standard) and ventilated critically ill patients (ICU Ventilated). We chose to split these two types of ICU wards to enable capacity planning on the required number of ventilators. Our model makes no distinction between discharge or death, as it has no consequences for capacity planning.

**Fig. 1:**
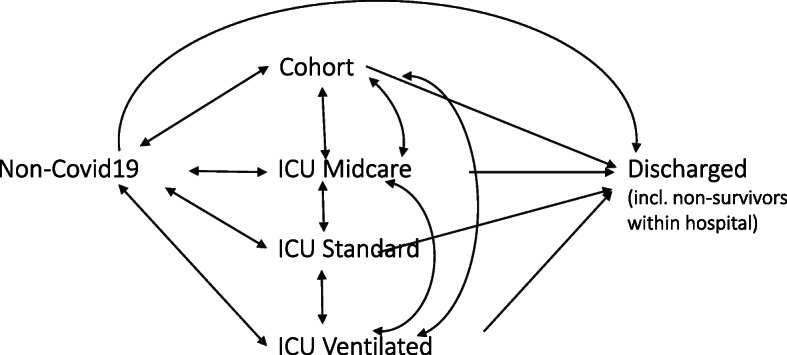
Possible transitions in the multistate model

Multistate models describe events over the course of time as transitions between multiple states. A first step is to define all possible transitions (Fig. 1, Appendix A1). Patients arrive at Non-Covid19 (e.g. emergency department), from which they can be transferred to Cohort (= all non-ICU wards with COVID-19 patients), ICU Midcare, ICU Standard or ICU Ventilated. Each patient can have multiple transfers between the different wards, terminating in state Discharged, which indicates that the patient either has been discharged or has died.

The considered multistate model places no constraints on the possible transitions that can be made. In particular, the cause-specific hazard of each transition is modelled non-parametrically and estimated using the Aalen-Johansen estimator [17]. This is done under a standard Markovian assumption that the hazard to transition to a given state (e.g. ward), while possibly different depending on the current state in which the patient is present, has no residual dependence on earlier states in which the patient was observed. For instance, the cause-specific hazard to transition to Cohort after having spent 10 days on ventilation is assumed to be the same, regardless of whether the patient was already in the ICU prior to ventilation, versus was directly admitted to ICU Ventilated. To enable a fast implementation and because covariate data of future cases are obviously missing, no covariate adjustment is made in these models.

All models were fitted in R (version 3.6.1), using the implementation from the *mstate* [18] package for multistate models (see Appendix A2).

### Simulating transition for patients already present in the hospital

The fitted multistate model was used to simulate on each day of the pandemic, how COVID-19 patients currently present in the hospital are expected to transition to other wards, discharge or death. In particular, for each patient, we calculated their cause-specific hazard to transition to each of the other wards, discharge or death on each of the subsequent 10 days, based on their latest state and the time already spent in that state. Based on these estimated cause-specific hazards, the patient’s possible transitions through the different states were then randomly simulated. Subsequently, the number of occupied beds on each ward was calculated for each of the subsequent 10 days. This process was repeated M times (in our case 500) in order to eliminate simulation error as well as to develop insight into the degree of uncertainty. Simulations were based on the R function *mstate::mssample* (see Appendix A2.4).

### Simulating transitions for new patients

To predict the number of new cases expected over the subsequent 10 day(s), we use additive Poisson modelling. In particular, we model the logarithm of the number of daily new cases using a Poisson model with a penalized regression spline for calendar time. Smoothing parameters are selected based on Mallow’s Cp. All models are fitted in R (version 3.6.1), using the implementation from the *mgcv* [19] package for additive Poisson modelling (Appendix A4).

Based on the fitted Poisson model, we next simulate the number of new cases that is expected to arrive on each of the coming 10 days. For convenience, these patients are assumed to enter the Non-Covid19 ward (such as ER) (R package *mgcv::gam*), with their time set to zero. Next, the fitted multistate model is used to simulate how new cases will transition to different wards, discharge or death over the coming 10 days. Also this entire simulation process was repeated five hundred times. In doing so, we accounted for the fact that e.g. for a patient who was simulated to be newly admitted on day eight, we only need to simulate his/her transitions for the subsequent two days (see Table 1).

**Table 1:** Example table of the predictions made for each day the next coming ten days

		1	2	3	4	5	6	7	8	9	10
		April 20, 2020	April 21, 2020	April 22, 2020	April 23, 2020	April 24, 2020	April 25, 2020	April 26, 2020	April 27, 2020	April 28, 2020	April 29, 2020
**1**	**April 20, 2020**	X									
**2**	**April 21, 2020**	X	x								
**3**	**April 22, 2020**	X	x	x							
**4**	**April 23, 2020**	X	x	x	x						
**5**	**April 24, 2020**	X	x	x	x	X					
**6**	**April 25, 2020**	X	x	x	x	X	x				
**7**	**April 26, 2020**	X	x	x	x	X	x	x			
**8**	**April 27, 2020**	X	x	x	x	X	x	x	x		
**9**	**April 28, 2020**	X	x	x	x	X	x	x	x	x	
**10**	**April 29, 2020**	X	x	x	x	X	x	x	x	x	x

Legend: An x represents 500 simulations in our analysis

The total number of occupied beds across existing and new patients was calculated for each of the subsequent 10 days in each of the M simulation runs. The obtained results were averaged across the M simulation runs to eliminate simulation error. In addition, to summarize the uncertainty in the possible capacity needed on each day, we report a best case scenario (corresponding to the 5% percentile of the needed capacity) and a worst scenario (corresponding to the 95% percentile of the needed capacity).

To assess the degree of inaccuracy in the results stemming from the limited number of simulation runs, we calculated Monte Carlo simulation error. For the mean scenario, this is given by the standard error of the mean (defined as the standard deviation of the capacity across the M simulations, divided by the square root of the number of simulations). For the two percentiles, we report the standard error calculated using Nyblom’s interpolated order statistic approach [20] (available from the R package *quantileCI::quantile_confint_nyblom*) (Appendix A3).

### Model validation

To validate the model we compare the bed occupancy predicted on April 20, 2020 and April 27, 2020, which corresponds with the first peak of the pandemic, with the actual values. We also evaluate one-day-ahead predictions in the period in between these two dates. This means that for every day a new prediction is made for just the next day, e.g. on April 23, 2020 the prediction is made for April 24, 2020 and on April 24, 2020 the prediction is made for April 25, 2020.

## Results

### Patient characteristics

We use and apply this approach on data from the Ghent University hospital during the COVID-19 pandemic. On April 20, 2020 this dataset consists of 203 different people in hospital, while on April 27, 2020 222 admissions are included. More males than females are admitted with an average age of sixty (Table 2). At the two selected time points, a large fraction of all patients (29–24%) is still in hospital.

**Table 2:** Patient characteristics for April 20, 2020 and April 27, 2020

	April 20, 2020	April 27, 2020
N (number included admissions)	203	222
Gender (male)	130 (64%)	139 (63%)
Age [SD]	56.84 [17.51]	57.07 [17.72]
Patient status at moment of data collection		
Non-survivors	21 (10%)	24 (11%)
Discharged	123 (61%)	145 (65%)
Non-discharged	59 (29%)	53 (24%)

Legend: *SD* Standard Deviation

### Multistate models

**Fig. 2:**
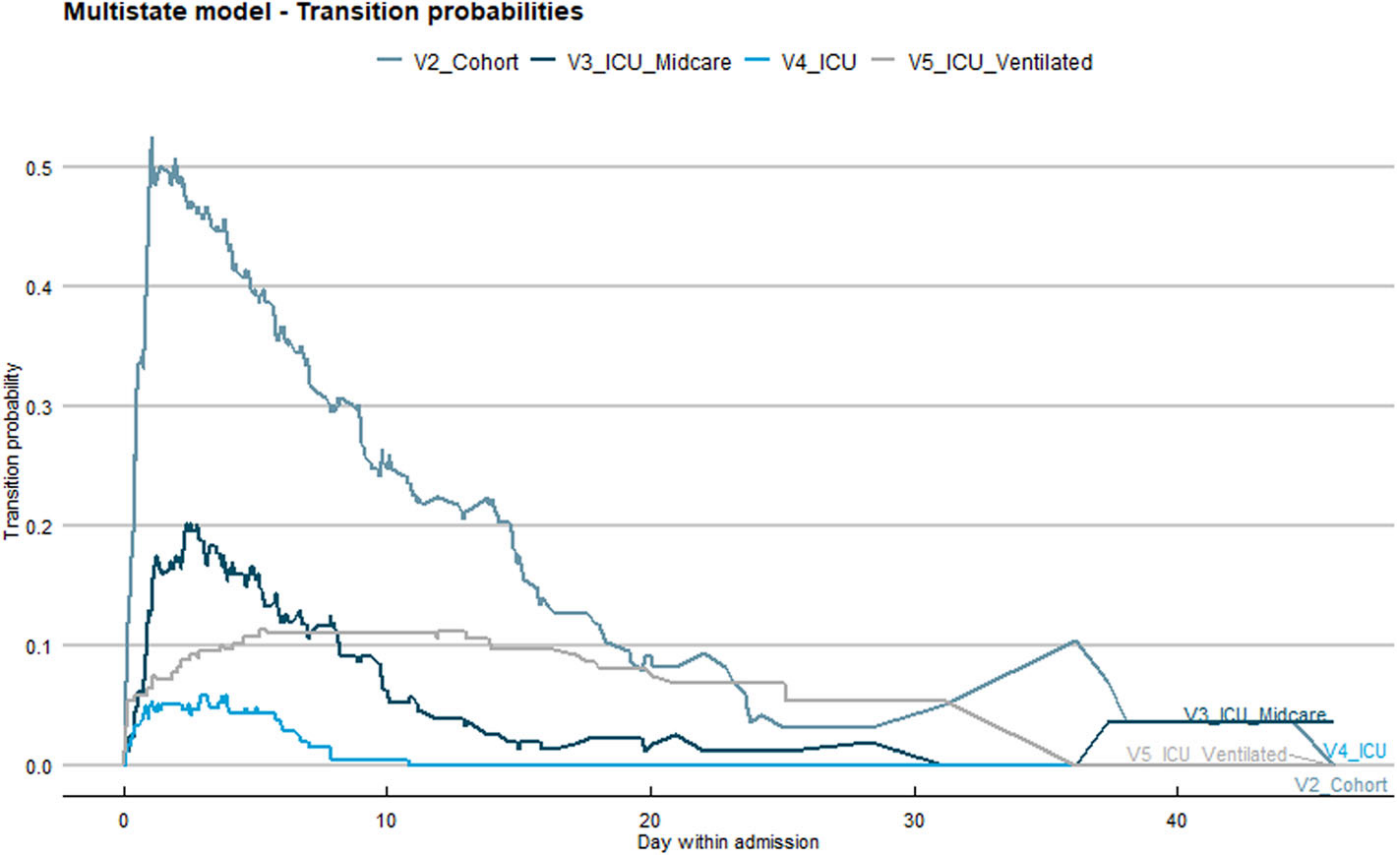
Transition probabilities: probabilities to transition from the first state (Non-Covid19) to selected states over the considered time window

We estimate the overall transition probabilities (using the *mstate::probtrans* function) from the multistate model (fit with *mstate::msfit*) (Appendix A1). In Fig. 2 we can see the overall transition probabilities for the ward in which we wish to predict the number of patients by day, which express what percentage of patients is expected in each state in function of the number of days since admission. It shows a majority of patients in Cohort, and moreover indicates long length of stay on ICU Ventilated.

### Simulating transition for all patients

**Fig. 3:**
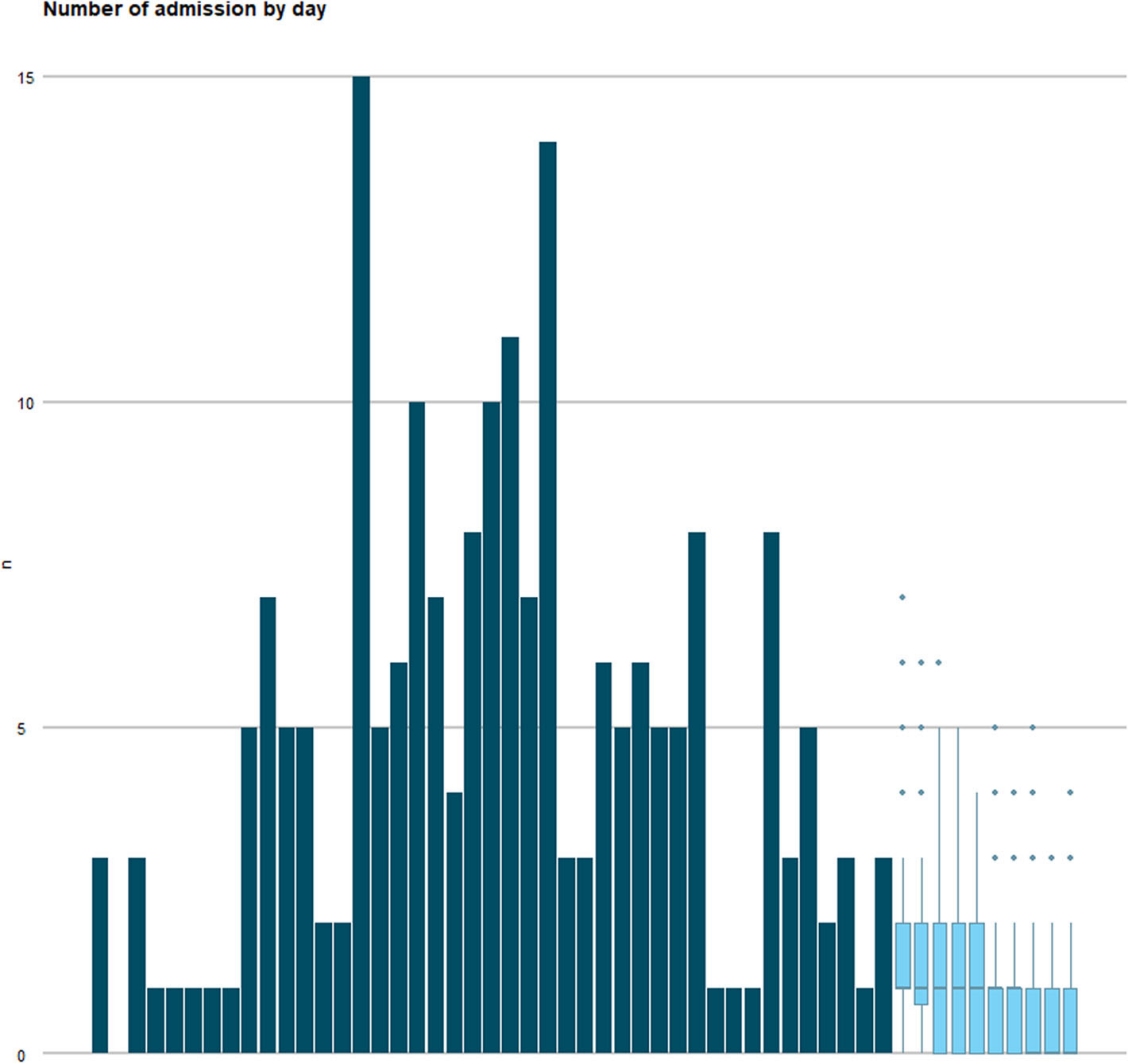
Observed and predicted number of new patients - Poisson modelling – April 20, 2020. The dark bars are the actual numbers of new admission by day. The lighter boxplots show the predicted numbers of expected admissions by day

The results of the Poisson modelling to predict the number of expected patients for the next ten days is given in Fig. 3. We show the actual numbers in dark bars and the predicted number of new patients in lighter boxplots. For interpretation of the boxplots we refer to Appendix A5.

**Fig. 4:**
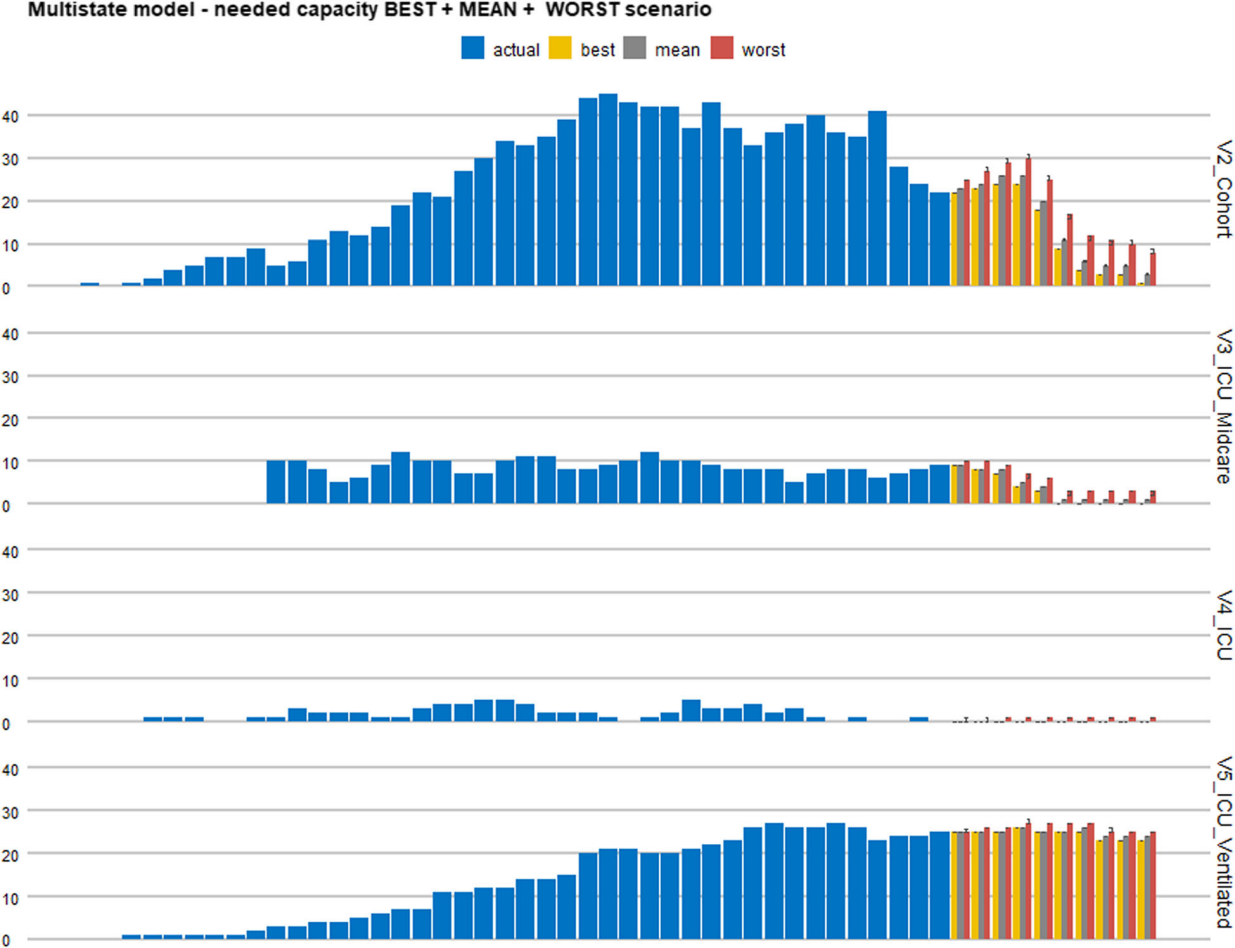
Resulting actual and predicted number of beds with the Monte Carlo simulation error for three scenarios: best (Q05), mean and worst (Q95) – April 20, 2020. There are four types of ward: Cohort, ICU Midcare, ICU Standard (=non-ventilated), ICU Ventilated

The results of the simulations are visualized in bar charts and in a table with the absolute numbers of patients. The bar chart (Fig. 4) shows the trends as well as the difference between actual and predicted number of patients; the table (Table 3) holds the absolute numbers which are of interest to stakeholders. The graph and table also display simulation error, which is small, indicating that 500 simulation runs suffice. As expected from the transition probabilities, the largest proportion of patients is expected to stay in Cohort. This number first increases, as this is where new patients arrive before possible transfer to other wards. We observe a similar effect on ICU Midcare, where transfers from Cohort and ICU Ventilated result in an increase of patients on this ward and transfers back or to these same wards for a decrease. The evolution on ICU Ventilated is different, with patients tending to have long lengths of stay once admitted. ICU Standard has a very limited number of patients.

**Table 3:** Result table

day	Cohort			ICU Midcare			ICU Standard			ICU Ventilated		
		Actual			Actual			Actual			Actual	
2020-04-15	36			8			0			27		
2020-04-16	35			8			1			26		
2020-04-17	41			6			0			23		
2020-04-18	28			7			0			24		
2020-04-19	24			8			1			24		
	**Best**	**Median**	**Worst**	**Best**	**Median**	**Worst**	**Best**	**Median**	**Worst**	**Best**	**Median**	**Worst**
2020-04 − 20	24 [24–24]	25 [24.96–25.04]	27 [27–28]	10 [10–10]	10 [9.98–10.02]	11 [10.6–11]	1 [1–1]	1 [0.98–1.02]	1 [1–1]	24 [24–24]	24 [23.98–24.02]	24 [24–26.13]
2020-04-21	25 [25–26]	27 [26.91–27.09]	30 [29–32]	8 [8–8]	9 [8.96–9.04]	10 [10–10.53]	1 [1–1]	1 [0.96–1.04]	1 [1–3.32]	24 [24–24]	24 [23.96–24.04]	25 [24.47–25.67]
2020-04-22	27 [26.89–28]	30 [29.84–30.16]	33 [32–33.11]	6 [6–6]	7 [6.93–7.07]	8 [8–9]	1 [1–1]	1 [0.93–1.07]	1 [1–2]	25 [25–25]	25 [24.93–25.07]	26 [26–27]
2020-04-23	31 [31–32]	34 [33.8–34.2]	37 [37–39.33]	5 [5–5]	6 [5.91–6.09]	8 [8–9]	1 [1–1]	1 [0.91–1.09]	1 [1–2]	26 [26–26]	26 [25.91–26.09]	27 [27–28]
2020-04-24	27 [26–27]	30 [29.76–30.24]	34 [33–35.22]	4 [4–4]	5 [4.89–5.11]	7 [7–8]	1 [1–1]	1 [0.89–1.11]	2 [1–2]	26 [26–26]	26 [25.89–26.11]	28 [28–28]
2020-04-25	23 [21.89–23.1]	26 [25.72–26.28]	30 [29–31.22]	2 [2–2]	3 [2.88–3.12]	6 [5–6]	1 [1–1]	1 [0.88–1.12]	2 [2–2]	25 [25–25]	26 [25.88–26.12]	27 [27–27.18]
2020-04-26	15 [14–16]	19 [18.7–19.3]	23 [22–25]	1 [1–1]	3 [2.86–3.14]	5 [5–6]	1 [1–1]	1 [0.86–1.14]	2 [1–2]	25 [25–25]	26 [25.86–26.14]	27 [27–28]
2020-04-27	9 [8–9]	12 [11.7–12.3]	16 [15.9–18]	1 [1–1]	3 [2.87–3.13]	5 [5–5]	1 [1–1]	1 [0.87–1.13]	2 [1.19–2]	25 [25–25]	26 [25.87–26.13]	28 [27–28]
2020-04-28	11 [9–11]	14 [13.7–14.3]	18 [17–20]	1 [1–1]	3 [2.87–3.13]	5 [5–6]	1 [1–1]	1 [0.87–1.13]	2 [1–2]	23 [23–23]	24 [23.87–24.13]	26 [25.46–26]
2020-04-29	9 [8–10]	13 [12.66–13.34]	17 [16–18.11]	1 [1–1]	3 [2.85–3.15]	5 [5–5]	1 [1–1]	1 [0.85–1.15]	2 [1–2]	22 [22–22]	23 [22.85–23.15]	25 [25–26]

Legend: Actual numbers of patients in the hospital at the specific wards and the predicted numbers for the next ten days – April 20, 2020. The predictions are made for the three different scenarios (Best Q05, Median. Worst Q90) with the Monte Carlo simulation error

#### Model validation

To validate our model, we compare the actual and predicted numbers of patients (Table 4).

**Table 4:** For each ward the prediction as on April 20, 2020 and April 27, 2020, for the next ten days, as reported via a best-mean-worst case scenario

day	Cohort			ICU Midcare			ICU Standard			ICU Ventilated		
	Predictions		Actual	Predictions		Actual	Predictions		Actual	Predictions		Actual
	20-apr	27-apr		20-apr	27-apr		20-apr	27-apr		20-apr	27-apr	
**April 15, 2020**			36			8			0			26
**April 16, 2020**			35			8			1			25
**April 17, 2020**			41			6			0			22
**April 18, 2020**			28			7			0			23
**April 19, 2020**			24			8			1			23
**April 20, 2020**	24–25 - 27		22	10–10 – 11		9	1–1 – 1		0	24–24 - 24		24
**April 21, 2020**	26–28 - 31		19	8–9 – 10		9	1–1 – 1		1	24–24 - 25		23
**April 22, 2020**	28–31 - 34		20	6–7 – 8		4	1–1 – 1		1	25–25 - 26		24
**April 23, 2020**	29–32 - 36		19	5–6 – 8		4	1–1 – 1		1	26–26 - 27		21
**April 24, 2020**	26–29 - 33		16	4–5 – 7		5	1–1 – 2		2	26–26 - 28		21
**April 25, 2020**	20–23 - 27		14	2–3 – 6		6	1–1 – 2		2	25–26 - 27		20
**April 26, 2020**	14–18 - 22		13	1–3 – 5		8	1–1 – 2		2	25–26 - 27		20
**April 27, 2020**	8–12 - 16		14	1–3 – 5		9	1–1 – 2		1	25–26 - 28		20
**April 28, 2020**	10–13 - 18	15–16 - 17	18	1–3 – 5	11–11 - 12	7	1–1 – 2	2–2 – 2	0	23–24 - 26	20–20 - 20	15
**April 29, 2020**	11–15 - 19	16–18 - 20	17	1–3 – 5	10–10 – 11	8	1–1 – 2	2–2 – 2	1	22–23 - 25	20–20 - 21	14
**April 30, 2020**		18–20 - 23	17		10–10 – 11	6		1–1 – 2	1		20–20 - 21	11
**May 1, 2020**		20–23 - 26	20		7–8 - 10	6		1–1 – 2	1		21–21 - 22	10
**May 2, 2020**		17–20 - 23	20		9–10 - 12	8		1–1 – 2	1		18–18 - 20	9
**May 3, 2020**		19–22 - 26	21		5–6 - 9	9		1–1 – 2	1		19–20 - 21	9
**May 4, 2020**		15–19 - 22	17		3–4 - 7	8		1–1 – 2	1		18–19 - 21	9
**May 5, 2020**		7–10 - 14	16		2–4 - 6	9		1–1 – 2	0		23–24 - 26	6
**May 6, 2020**		6–9 - 13	15		3–5 - 7	8		1–1 – 2	1		17–18 - 20	6
**May 7, 2020**		6–10 - 14	16		4–6 - 8	9		1–1 – 2	1		17–18 - 21	5

Legend: In the `Actual’ column we show the number of actual used beds in the hospital (at noon)

Where Table 4 shows the prediction for the next 10 days, Table 5 shows the prediction for just the next day. If the actual difference on one day is large, this has a negative effect on the prediction (e.g. April 22, 2020 ICU Midcare). This has no effect on the prediction for the days after, as the actual numbers are included in the dataset. Nevertheless, this does imply that for prediction on multiple days (e.g. 10 days as in Table 4) will results in an unexpected change that will be lower or higher than expected.

**Table 5:** One-day-ahead predictions - mean case scenario

	Cohort			ICU Midcare			ICU Standard			ICU Ventilated		
actual day	Actual	prediction	diff	actual	prediction	diff	actual	prediction	diff	actual	prediction	Diff
**April 20, 2020**	22			9			0			25		
**April 21, 2020**	19	23	4	9	9	0	1	0	-1	24	25	1
**April 22, 2020**	20	21	1	4	9	5	1	0	-1	25	24	-1
**April 23, 2020**	19	21	2	4	3	-1	1	1	0	22	25	3
**April 24, 2020**	16	20	4	5	4	-1	2	1	-1	22	21	-1
**April 25, 2020**	14	19	5	6	4	-2	2	2	0	21	21	0
**April 26, 2020**	13	16	3	9	6	−3	2	2	0	20	20	0
**April 27, 2020**	14	14	0	10	8	−2	1	2	1	20	20	0
**April 28, 2020**	17	15	−2	8	10	2	0	1	1	15	20	5

Legend: `Actual’ refers to the actual numbers of patients on the actual day. `Prediction’ refers to the prediction number of patients for this Ward type made on the previous day. `Diff’ shows the difference between the predicted and the actual number of patients

The large difference on April 28, 2020 in ICU Ventilated is caused by a sudden two deaths and three transfers.

## Discussion

In this paper, we have proposed an algorithm that can be used for capacity planning during an epidemic, along with software code. We are not aware of similar data driven approaches that fully rely on one’s own hospital health records.

We have found the proposed approach to be fairly reliable in predicting the required capacity within Ghent University hospital, except at the start of the pandemic where the number of data is still too limited to enable reliable prediction, and where the organization may not be in a sufficiently stable situation to enable extrapolation to the future. For this, it can be useful to borrow strength by combining data across multiple regional hospitals. Also, it can be used in a second wave, using the parameters from the first wave. This would then become more specific for the institution, as physicians and treatment protocols differ among the different institutions.

While using this tool during the COVID-19 pandemic, we have found the number of patients on ICU Midcare to be the hardest to predict (Table 5), where we observe between − 3 and + 5 deviation between the actual and predicted numbers. The reason is that this ward receives input and output from Cohort as from ICU Standard/ICU Ventilated, making the numbers of patients on this ward very sensitive to human decisions made on these other wards, and possibly even the insight of a single physician. Our results, including the ones reported in this paper (and used by the task force of the Ghent University hospital), are based on predictions made on Mondays. These are subject to a possible weekend effect, as decisions on opening/closing/changing wards were usually made just prior to the weekend and this could influence the results. These decisions mainly related to shifting or changing ICU Midcare, adding to the difficulty of predicting the capacity on ICU Midcare on Mondays.

A further limitation of our proposal is that the Poisson model may need some time to pick up sudden increases or drops in the expected number of new cases, e.g. due to a relaxation of lockdown regulations. The use of smoothing splines allows sufficient flexibility to pick up such effects, but some time is needed for this to be picked up in a reliable way.

The proposed approach is simulation-based, which is useful to develop insight into random fluctuations that may occur in the required capacity. The calculated Monte Carlo simulation error suggested 500 simulations to suffice in order to dampen simulation error. Model validation was based on an independent dataset as the predictions were evaluated on future data not known upfront or used within the training set data.

Our reported best- and worst-case scenario ignore the excess variability that may arise from the fact that the Poisson and multistate models were themselves fitted on limited data and are thus subject to imprecision, making the reported intervals somewhat optimistic. Acknowledging this excess variability is non-trivial, and beyond the scope of this work.

The proposed approach is transposable to any other epidemic or very specific pathology/disease for which one is interested to know the required number of beds for specific wards and with a specific flow. As most administrative systems will have at least one row for every transfer for all patients, these can be bundled and as such used in the same approach as described. The extent of data manipulation should not be underestimated as each system has its own layout / structured and is difficult to generalize internationally (in Belgium most hospitals have one of the larger software vendors to capture this administrative data). We presume that this step will take most of the time to set up the model for the planning tool. The need for an accurate planning tool is high, as a lack of equipment to apply oxygen related therapies, such as invasive mechanical ventilation, increases mortality [21], and so does an overflow on ICU beds [22]. An optimal organization within the hospitals is therefore needed, and we believe that prediction strategies as discussed in this paper can be helpful to obtain this objective.

The prediction accuracy of our model can in principle be further improved by making use of patient characteristics, such as age and gender, when modelling transitions between wards. We have chosen not to do this in view of the additional cost of data manipulation, the risk of model misspecification when Cox proportional hazards models are used for the cause-specific transition hazards, and the fact that such patient characteristics are unknown for future patients.

## Conclusions

The proposed algorithm can be quickly setup and is an added value during the COVID-19 pandemic to predict the needed capacity within the hospital by ward type.

## Supplementary Information

**Additional file 1.**

## Data Availability

The data (csv file) that support the findings of this study are available from https://github.com/descheppermieke/Prediction-of-hospital-bed-capacity-during-the-COVID-19-pandemic.

## Abbreviations

ICU: Intensive Care Unit
PCR: Polymerase chain reaction
