# Supplementary material for: Prediction of hospital bed capacity during the COVID− 19 pandemic
Source: BMC Health Serv Res. 2021 May 18;21:468. doi: 10.1186/s12913-021-06492-3 (PMC8128685; doi:10.1186/s12913-021-06492-3)
Supplement: Supplementary file 1 — Additional file 1. [file 12913_2021_6492_MOESM1_ESM.pdf]

Appendix - Prediction of hospital bed capacity during the COVID-19 pandemic

Mieke Deschepper [a], Kristof Eeckloo [a,b], Simon Malfait [a], Dominique Benoit [c], Steven Callens [d], Stijn Vansteelandt [e,f]

a Strategic Policy Cell at Ghent University Hospital, C. Heymanslaan 10, 9000 Ghent, Belgium
b Department of Public Health and Primary Care, Ghent University, C. Heymanslaan 10, 9000 Ghent
c Department of Intensive Care medicine, Ghent University Hospital, C. Heymanslaan 10, 9000 Ghent
d Department of General Internal Medicine, Ghent University Hospital, , C. Heymanslaan 10, 9000 Ghent, Belgium
e Department of Applied Mathematics, Computer Science and Statistics, Ghent University, Krijgslaan 281 S9, 9000 Ghent
f Department of Medical Statistics, London School of Hygiene and Tropical Medicine, Keppel Street, WC1E 7HT London

This appendix for the article *Prediction of hospital bed capacity during the COVID-19 pandemic* hold the implementation of the different steps as written in the methods and result section. Among others, one can find code snippets how to handle the problem.

A1 How to build a transition Matrix using *mstate::tmat*

```
tmat <- transMat(x = list(c(2,3,4,5,6),           #non covid19 =1
                          c(1,3,4,5,6),  #cohort=2
                          c(1,2,4,5,6),  #icu midcare=3
                          c(1,2,3,5,6),  #icu standard=4
                          c(1,2,3,4,6),  #icu ventilated=5
                          c()),          #discharge=6
                 names=namen_type_vpe)
```

The transition matrix (Figure 1) has 25 possible transition (Table A1.1).

Table A1.1 Transition Matrix (6 states, 25 transitions)

	**Non-Covid19**	**Cohort**	**ICU Midcare**	**ICU Standard**	**ICU Ventilated**	**Discharge**
Non-Covid19	NA	1	2	3	4	5
Cohort	6	NA	7	8	9	10
ICU Midcare	11	12	NA	13	14	15
ICU Standard	16	17	18	NA	19	20
ICU Ventilated	21	22	23	24	NA	25
Discharge	NA	NA	NA	NA	NA	NA

A2 Data preprocessing

A2.1 Setup the data to the input format for *mstate*

An important and time consuming step in the setup of this model is the data input for the multistate model.

Before starting the simulation, we need to format the data as expected for the *mstate* R package. This package foresees in an easy use for data manipulation if the data is already in a row format as it would be for a survival analysis. This is not the case for our data, which has rows for every transfer for all patients.

The format to use has at least the columns: id (unique identifier), from (ward where patients stays during this period), to (ward where the patient will be transferred to), trans (transition number of the transition matrix), Tstart (start time), Tstop (end time), time (cumulative time), and status (1 if event has occurred, 0 if censored).

For our dataset we perform three steps, First, combine all rows which have the same type of ward and calculate a new start and end time in order to recalculate the length of stay within this period. Second, calculate the cumulative length of stay for the patients and define the "to" ward. When the patient is still present in the hospital, the last line for this patient is stated as discharged but with a status zero (censoring). Last, the data is filled with status zero lines for all other possible transitions for each period.

A2.2 Setup the multistate model using *mstate::msfit*

Once the data is in the desired (long) format one can start modelling. First we estimate the cumulative hazards Next, we use these cumulative hazard to estimate the transition hazards, which needs the transition matrix as input (using *mstate::msfit*).

```
## estimate cumultaive hazards
c0 <- coxph(Surv(Tstart,Tstop,status) ~ strata(trans),
             data=ds_result,
             method="breslow")

## estimate transition hazards
msf0 <- msfit(c0,
             trans=tmat,
             vartype = "greenwood",
             variance = FALSE)
```

A2.3 Compute the overall transition probabilities of the multistate model using *mstate::probtrans*

Optionally the overall transition probabilities in the multistate can be computed to visualize the model using *mstate::probtrans*. These transition probabilities show in which state (in our case a ward) the patient probably will be on each day in the hospital.

```
pt0 <- probtrans(msf0,predt=0,method="greenwood")
res_prob <- pt0[[1]] %>%
             select(time, starts_with("pstate")) %>%
             pivot_longer(cols= c(-time),
                          names_to = "pstate",
```

```
                                values_to = "trans_prob")
```

A2.4 Simulating transition for patients already present in the hospital using *mstate::mssample*

To start the simulation we need for patients present in the hospital their latest state, as a number of the transition matrix, and the time already in this state. For example patient A is at this moment on Cohort already for 26 hours, than the state will be 2 and the time is 26.

To simulate the transitions we use *mstate::mssample* and is performed M times (in our case 500). Finally, we need to map the output to the actual day of admission.

```
histlist <- list(state=currentpat$state[i], time= currentpat$time[i])
sim_res <- mssample(Haz=msf0$Haz,
                      trans=tmat,
                      tvec=unique(msf0$Haz$time),
                      history = histlist,
                      output = "data",
                      M=500)

sim_output <- map_df(sim_res, unlist) %>%
    filter(status == 1) %>%
    mutate(toDagVanafVandaag = Tstop - Tstart[1],
           fromDagVanafVandaag = Tstart - Tstart[1])
```

A3 Predict number of new patients using *mgcv::gam*

```
model_day <- gam(formula = n ~ s(as.numeric(day)),
             family = poisson,
             data = head(ds_by_day,-1))# be sure that the data contains 0 if no
                                      # patients are admitted at a certain day,
                                      # we leave out the last day, as this only
                                      # contains data until 8 a.m.


## predicton
p_day <- predict.gam(model_day, type = "response",
                      newdata = data.frame(day =
                  c(max(ds_by_day$day):(max(ds_by_day$day+nrToPredict-1)))))

## result simulation
sim_p_day <- map(p_day, ~(rpois(lambda= .x, n = nrToPredict)))
```

A4 Simulating transitions for new patients and calculate standard error using Nyblom's interpolated order statistic approach using quantileCI::quantile_confint_nyblom

```
newpat_sum <- result_predictions_new_patients %>%
  group_by(day, state) %>%
  summarise(
    Min = min(n),
    Q25 = quantile(n, 0.25),
    Mean = mean(n),
    Median = median(n),
    Q75 = quantile(n, 0.75),
    Q95 = quantile(n, 0.95),
    Max = max(n),
    Q05_low =(quantileCI::quantile_confint_nyblom(x=n,
              p=0.05, conf.level=0.95,interpolate=TRUE)[1]),
    Q05_high =(quantileCI::quantile_confint_nyblom(x=n,
              p=0.05, conf.level=0.95,interpolate=TRUE)[2]),
    Q95_low =(quantileCI::quantile_confint_nyblom(x=n,
              p=0.95, conf.level=0.95,interpolate=TRUE)[1]),
    Q95_high =(quantileCI::quantile_confint_nyblom(x=n,
              p=0.95, conf.level=0.95,interpolate=TRUE)[2]),
    Median_low =(quantileCI::quantile_confint_nyblom(x=n,
              p=0.50, conf.level=0.95,interpolate=TRUE)[1]),
    Median_high =(quantileCI::quantile_confint_nyblom(x=n,
              p=0.50, conf.level=0.95,interpolate=TRUE)[2])))

  if(scenario== "worst"){
    np <- newpat_sum %>%
      mutate(n=round(Q95),
             n_high=(Q95_high),
             n_low=(Q95_low)) %>%
      mutate(state = as.character(state)) %>%
      select(day, state, n, n_high, n_low)
  }else if(scenario=="median"){
    np <- newpat_sum %>% rename(n=Median) %>%
      mutate( n_high=(Median_high),
              n_low=(Median_low),
        state = as.character(state)) %>%
      select(day, state, n, n_high, n_low)
  }else if(scenario=="mean"){
    np <- newpat_sum %>%
      mutate(n=round(Mean)) %>%
      mutate(state = as.character(state)) %>%
      select(day, state, n) %>%
    mutate(n_high = (n+ sd(n)/sqrt(nrToPredict)),
           n_low = (n - sd(n)/sqrt(nrToPredict)))
  }else if(scenario=="best"){
    np <- newpat_sum %>%
      mutate(n=round(Q05),
             n_high=(Q05_high),
             n_low=(Q05_low)) %>%
      mutate(state = as.character(state)) %>%
      select(day, state, n, n_high, n_low)
  }
```

A5 Boxplot interpretation

In Figure A5.1 we show how one can interpret a boxplot.

The blue box shows 50% of all results. The middle line is the median, the upper line of the box is the 75th percentile and the lower line of the box is the 25th percentile. The highest point of the whisker is the maximum, the lowest point of the whisker is the minimum. If there are dots above or below the whiskers, than we indicate these as outliers.

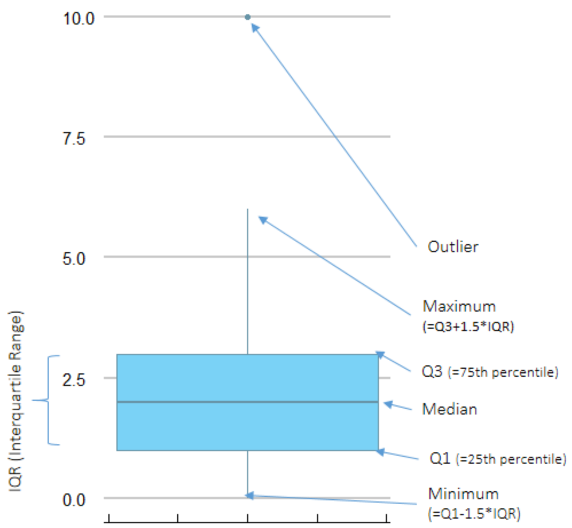

Figure A5.1 How to interpret a boxplot
